# Genetic parameters and selection response for the harvest body weight of the giant freshwater prawn (*Macrobrachium rosenbergii*) in a breeding program in China

**DOI:** 10.1371/journal.pone.0218379

**Published:** 2019-08-12

**Authors:** Juan Sui, Sheng Luan, Guoliang Yang, Zhenglong Xia, Kun Luo, Qiongying Tang, Xia Lu, Xianhong Meng, Jie Kong

**Affiliations:** 1 Key Laboratory for Sustainable Utilization of Marine Fisheries Resources, Ministry of Agriculture, Yellow Sea Fisheries Research Institute, Chinese Academy of Fishery Sciences, Qingdao, China; 2 Laboratory for Marine Fisheries Science and Food Production Processes, Qingdao National Laboratory for Marine Science and Technology, Qingdao, China; 3 Huzhou University, Huzhou, China; 4 Jiangsu Shufeng Prawn Breeding Co. LTD, Gaoyou, China; Shanghai Ocean University, CHINA

## Abstract

A multi-trait selective breeding program of *Macrobrachium rosenbergii* was initiated in China in 2015. In this program, the *M*. *rosenbergii* resources were widely collected from four countries, the origin of the founders was verified with 16 microsatellites and the pedigree was reconstructed, and the optimum contribution selection was used to make the mating design. In this study, we evaluated the genetic parameters and selection response for the harvest body weight (HBW) of *M*. *rosenbergii* after being communally reared for 95–109 days. The data were collected from two generations that comprised 25,212 progenies from 150 sires and 198 dams. The residual maximum-likelihood methodology was employed to evaluate the variance components, by fitting an animal model. The accuracy of estimated breeding values increased by 0.38% after pedigree reconstruction using microsatellite markers. The estimated heritability (*h*^*2*^) for HBW was moderate (0.212 ± 0.049) and the common environmental coefficient (*c*^*2*^) was low (0.063 ± 0.017) when all the data were used for the analysis. Within generations, *h*^*2*^ was moderate to high (0.198 ± 0.080 to 0.338 ± 0.049). *c*^*2*^ could only be estimated in G_1_, which was 0.055 ± 0.030. The average HBW of males was significantly larger than that of females (*P* < 0.01). *h*^*2*^ estimated for female HBWs were higher than that for males within generations, while *h*^*2*^ estimated for female HBWs were lower than that for males across generations. But they were not significantly different (*P* > 0.05). The genetic correlations between sexes were moderate to high within each generation (0.529 to 0.763). Two methods were used to estimate the realized response. One method was calculated from the differences between the least squares means of the selected population HBW and that of control population HBW, which was 14.01%. The other method was calculated from the differences between the EBVs of the selected population HBW and that of control population HBW, which was 11.52%. The predicted responses derived from two sets of genetic parameters acquired from within- and across- generation datasets were 11.68% and 10.67%, respectively. The present study provides valuable information for breeding programs of *M*. *rosenbergii*.

## Introduction

The giant freshwater prawn, *Macrobrachium rosenbergii*, is a species with great economic value in the world. In 2016, its production reached 0.23 million tons globally, with a value of more than 1.90 billion US dollars. China is an important producer of *M*. *rosenbergii*. China’s production was over 0.13 million tons in 2016, accounting for more than half of the global production [1]. Nevertheless, *M*. *rosenbergii* is not a native species in China, most cultured populations are wild broodstocks introduced from other Southeast Asia countries, or selected populations with narrow genetic variation. There would be a risk of introducing pathogens and inbreeding, if using such populations over the long term.

Selective breeding could effectively improve the production traits of farmed animals and help the animals adapt to local conditions. In aquaculture, selective breeding has been used to improve growth, disease resistance, or quality traits in a lot of species, like Atlantic salmon (*Salmo salar*), rohu carp (*Labeo rohita)*, Nile tilapia (*Oreochromis niloticus*), and pacific white shrimp (*Litopenaeus vannamei*), with great success [2]. The selective breeding programs for *M*. *rosenbergii* have been initiated in several countries, such as China, India, and Vietnam [3–7]. In China, a selective breeding program aimed at improving harvest body weight (HBW) and pond survival of *M*. *rosenbergii* was initiated in 2006. A newly selected breed named “Nantaihu No. 2” was approved by the National Certification Committee for Aquatic Varieties of China in 2009 (registration no. GS01-001-2009). The new breed brought remarkable economic and social benefits. However, for the previous breeding program of *M*. *rosenbergii*, the founder population was generally considered to have no genetic linkage, which was often not the case. Also, assortative mating design (good families mated with good families and good individuals mated with good individuals), was a common mating method to produce the next generation; however, it may not be the best method. Truncation selection based on breeding values may result in inbreeding accumulation in progeny [8, 9].

Implementing a breeding program of *M*. *rosenbergii* with a faster growth rate and high pond survival is in urgent demand in China. Owing to this demand, another selection breeding program was initiated in 2015; firstly, the *M*. *rosenbergii* resources were widely collected from different countries; secondly, the molecular pedigree of founders based on microsatellite markers were used in this breeding program, which was successfully applied to avoid inbreeding in other aquatic species [10–12]; thirdly, the optimum contribution selection was used to make the mating design, to maximize long-term genetic progress and control inbreeding simultaneously [13, 14]. Up to now, the program has achieved remarkable progress, and is still being developed further.

The aim of the current study was to evaluate the HBW heritability, the accuracy of the breeding value, and the genetic correlations between males and females, as well as the selection responses after applying these improvements. The results would provide valuable information for the breeding of *M*. *rosenbergii*, as well as other aquatic animals.

## Materials and methods

### The genetic material

The founder population involved four different strains: The first strain was the progeny of the selected population “Nantaihu No. 2” in China [3], which was characterized by fast growth and high pond survival. There was likely some genetic connection among these individuals. The second strain was commercial larvae from Thailand Charoen Pokphand Group, characterized by fast growth in late stages. The last two strains were the progeny of wild populations from Burma and Bengal. A total of 134 prawns (56 females and 78 males) were used to construct the base population. All these strains were collected in 2015 and bred in Jiangsu Shufeng Prawn Breeding Industry Co., Ltd.

### DNA extraction, amplification, and pedigree reconstruction

Pleopods were collected from 134 founder broodstocks and stored in 95% alcohol. Genomic DNA was extracted by the standard phenol and chloroform method [15]. Primer sets for 58 dinucleotide microsatellite loci from previously published literatures [16–19] or developed in our laboratory were used for amplification of alleles. PCR reactions were carried out in a 20 μl reaction mixture, containing 25 ng of template DNA, 5 pmol of each primer, 1.5 mM of MgCl_2_, 200 μM of each dNTPs, 1X Taq buffer, and 0.25 U of Taq DNA polymerase (TAKARA). PCR amplification was performed in a BIO-RAD T100^™^ thermal cycler with the following cyclic conditions: initial denaturation at 94°C for 5 min, followed by 30 cycles at 94°C for 30 s, 30 s for annealing at a specific temperature (Table 1), 45 s for elongation at 72°C, and a final extension at 72°C for 5 min. The PCR products were genotyped by ABI 3730. A total of 16 microsatellite markers were selected (Table 1). The information of 16 loci in the 134 founders was shown in Table 2. The number of alleles per locus ranged from 7 to 22, with an average of 14 alleles per locus. The observed heterozygosity (H_O_) varied from 0.120 to 0.806 while the expected heterozygosity (H_E_) varied from 0.681 to 0.893. The polymorphic information content (PIC) varied from 0.630 to 0.879. The PIC measures were all larger than 0.5 in this study.

**Table 1:** List of microsatellite loci amplified in the breeding population of *M*. *rosenbergii*.

Locus name	Accession number	Repeat types	Primer 5′-3′	Allele range	Annealing temperature
HQ722928	HQ722928.1	(AG)_30_	F: GCTCATTCCGTCCTACATTCCTC R: GGGTACTTATCCCTCCCATTGC	189–350	64
HQ722929	HQ722929.1	(CT)_25_	F: CAGCTCTAACCTGATTGAAAGAC R: GCAGGCATTATCGTTACTTCTCC	225–262	55
HQ722930	HQ722930.1	(GA)_24_	F: GAAGACAATCGGCAACGAAAATA R: TCAGGGTGTAGTCTCTCGTTCTG	247–283	65
HQ722934	HQ722934.1	(TG)_11_	F: TTCCTGTGTGAATGTTGAGATGC R: GAGAACTTTCGGTTTACCCTTCC	232–260	57.6
HQ722935	HQ722935.1	(AG)_29_	F: GGAACAAATGAAGACTTGGATGC R: CTCTCTCTCCCTCAAGGTGTTGG	243–295	57.6
SUGbp8-137	EF204180	(ATT)_15_	F: CGACTGGGTGGTATTTAT R: CGCTGACGTTTATTCTGT	279–316	55
EMR31B	-	(CA)_7_	F: GCTGTGCTCCAAAATCTCTCTC R: CTCACCCATACTTGACAACGAC	196–239	58
Mbr-1	DQ019863	(GA)_24_	F: CCCACCATCAATTCTCACTTACC R: TCCTTTTCACATCGTTTCCAGTC	259–305	60
Mbr-3	DQ019865	(AG)_14_	F: CAACTCTATGTTTCGGCATTTGG R: GGGGAATTTTACCGATGTTTCTG	226–286	62
Mbr-4	DQ019866	(GT)_29_	F: CCACCTACCGTACATTCCCAAAC R: CGGGGCGACTTTTAGTATCGAC	216–300	62
Mbr-5	DQ019867	(AG)_25_	F: CAAGGCTCGTGTCTCTTGTTTC R: GCTTGTACTTGTTCAGCTTTTGC	288–326	62
Mbr-9	DQ019873	(TG)_5_(AG)_17_	F: TTGTTTGCTTGTTTAGTGTCAAGG R: CTCCAAAACCGAAAAATCCTCAC	229–272	60
MR5	-	(AG)_32_	F: GTAATGAACAGCACGAAAAGGAAG R: TCTGCGTTATTTTGAGTTTGGTATG	276–319	54
MR75	-	(AAG)_10_	F: AAGAGGATTTGGAGCGATTGG R: CTGAGTAAGATACGACGCCTTC	169–233	54
MR78	-	(GAA)_18_	F: TCTCATCTCCCCTCGTAGCATC R: TTATTTGTATTCATCTTCCGCCAT	214–298	54
MR146	-	(AAG)_17_	F: AAAGCCTGTTGAGAAGGATTGG R: GATTTGCTGGTGGAGAATTTGAG	230–307	54

**Table 2:** The information of 16 loci in the 134 founders.

Locus Name	N_A_	H_O_	H_E_	PIC	HW
HQ722928	22	0.403	0.866	0.849	***
HQ722929	11	0.604	0.867	0.849	**
HQ722930	13	0.224	0.739	0.709	***
HQ722934	7	0.500	0.681	0.630	***
HQ722935	15	0.612	0.857	0.840	NS
SUGbp8-137	11	0.418	0.766	0.739	***
EMR31B	15	0.120	0.893	0.879	ND
Mbr-1	19	0.392	0.858	0.842	***
Mbr-3	15	0.208	0.745	0.711	***
Mbr-4	21	0.672	0.782	0.764	NS
Mbr-5	13	0.624	0.858	0.839	NS
Mbr-9	13	0.496	0.842	0.823	***
MR5	15	0.436	0.808	0.785	***
MR75	12	0.806	0.841	0.821	NS
MR78	15	0.194	0.813	0.794	***
MR146	12	0.634	0.808	0.782	**
Mean	14	0.459	0.8139	0.791	-

N_A_: alleles per locus;

H_O_: observed proportion of heterozygosity;

H_E_: expected proportion of heterozygosity;

PIC: polymorphic information content;

HW: Hardy-Weinberg equilibrium. NS means no significance deviation; ND means not detected; *** means level of statistical significance deviation at *P*<0.001; ** means level of statistical significance deviation at *P*<0.05.

Cervus 3.0 software was used to carry out the identity analysis for the 134 founder individuals with the likelihood-based method and the information of the 16 loci [20]. COLONY 2.0 [21–23] as implemented in R [24], was used to infer full-sib relationships according to the genotypes of candidate parents. COLONY uses a Bayesian approach to reconstruct the most likely full-sib groups. This analysis was carried out using the full-likelihood method. The 134 founders were assigned to 110 families, with 56 sires from 54 families, and 78 dams from 64 families.

### Mating and production of families

G_0_ generation was established in 2016 by using an incomplete diallel crossing. The breeding design and the results of the family number for each breeding unit is shown in Table 3. Our previous experiments showed that the number of effective populations in the Bengal and Burma populations was too small, and the growth performance was always lower than that of the selected populations (Thailand and “Nantaihu No. 2”). Therefore, intra-population mating families and cross-breeding families between the two wild populations were no longer established. The survival rates of the two wild populations were better than that of the selected populations, so a certain number of hybrid families with other populations were designed. Similarly, the number of effective populations in Thailand is not large, so no intra-population mating families were established.

**Table 3:** The mating design and family number for each cross in G_0_.

Female	Male			
	Bengal	Burma	Thailand	“Nantaihu No. 2”
Bengal	-	-	7	6
Burma	-	-	8	5
Thailand	2	1	-	4
“Nantaihu No. 2”	4	5	8	28

A selection index was used to rank all harvest shrimps from G_0_ to G_1_. The relative weights were 70% for individual breeding values for harvest body weight (HBW), and 30% for family breeding values for pond survival. Herein, we focus on the genetic evaluation for the harvest body weights from G_0_ to G_1_. Pond survival was estimated using a standard threshold (probit) and sire-dam model, which will be described in detail in next paper for publication.

G_1_ generation was established by using an optimal contribution selection based on a selection index, with a rate of inbreeding of 0.5% [25]. 72 mating groups were designed to generate G_1_. Within each mating group, one male was mated to 3–5 females from different families. The control population was constructed using 15 full-sib families, whose parents had the mean selection index in the G_0_ generation and were from different full-sib families. Not all groups can be mated successfully according to the mating design. Sometimes only one pair could succeed in a group, which lead to the number of half-sib families being less than the number of candidate sires used in two generations.

Female and male candidate parents with a healthy appearance were selected, and each was reared in a cuboidal net cage with a side length of 0.25 m until fertilization. These cages were marked by a four-digit code, which was used as the individual ID. According to the mating design, 3–5 females and one male from different families were isolated and put in a separate 3 m^2^ tank. These candidates were identified by unique color combinations of “Visible Implant Fluorescent Elastomers” (VIE) for each family. Each female prawn carrying fertilized eggs was put into a separate 170 L tank until hatching. Two thousand larvae were randomly selected from each tank and put into a 50 L culture tank. The family establishment periods ranged from 32 to 35 days in G_0_ and G_1_ (Table 4). A total of 78 families were produced in G_0_ by mating 56 males and 78 females, and 120 families were produced in G_1_ by mating 90 males and 120 females (Table 5).

**Table 4:** Schedule of family reproduction and management for each generation.

Generation	Synchronization of family production			Average days for rearing separately	Ponds for rearing communally^a^	Stocking density/(ind·m^−2^)	Days for grow-out test			Harvest density/(ind·m^−2^)	Survival rate/%
	Start date (D/M/Y)	End date (D/M/Y)	Days				Stocking date (D/M/Y)	Harvesting date (D/M/Y)	Days		
G_0_	06/04/2016	11/05/2016	35	29–64	P11, P14	3.19; 3.29	02/07/2016	19/10/2016	109	2.35; 2.45	74; 74
G_1_	12/04/2017	14/05/2017	32	41–66	P10, P11	14; 9.75	06/07/2107	09/10/2017	95	6; 4.77	42.9; 48.9

^a^ P10:2133 m^2^; P11: 2000 m^2^; P14: 1933 m^2^

**Table 5:** Numbers of sires, dams, full-sib and half-sib families by generation.

Generation	Population	Sires	Dams	Full-sib families	Half-sib families
G_0_	Base	56	78	78	44
G_1_	Total	90	120	120	49
	Selection	80	105	105	47
	Control	14	15	15	2

### Rearing and family tagging

Larvae were fed twice a day with *Artemia salina* during the first 7 days after hatching. From the 8^th^ day, egg yolks was gradually added according to different developmental stages. Larvae were fed 4–8 times a day. Total amount and composition of the diet were adjusted daily according to different stages. The temperature was controlled at 30 ± 0.5°C. Daily water exchanges were gradually increased, reaching 100% at the post-larval stage.

Six hundred post larvae with strong vitality were randomly selected from each family, and put into a 3 m^2^ concrete tank until they grew to a body length of about 2.5 cm. Then the VIE tag was individually applied to identify each family. For G_0_, 150 prawns per family were randomly selected. For G_1_, 300 prawns per family were randomly selected. The selected prawns were assigned equally to each pond.

Pathogens, including *Enterobacter cloacae*, *Citrobacter freundii*, *Aeromonas hydrophila*, *Enterobacter aerogenes*, Nodavirus, Norovirus and Dicistrovirus, were detected by sampling and testing eggs and larvae at different stages from each family, using reverse transcriptase-polymerase chain reaction (RT-PCR). Families with pathogens were eliminated.

### Growth and harvest

Three earthen ponds were used for communally rearing two generations (Table 4), P11 (2000 m^2^) and P14 (1933 m^2^) were utilized in the G_0_ generation, and P10 (1933 m^2^) and P11 (2000 m^2^) were utilized in the G_1_ generation. The temperature was 25 ± 6°C during the grow-out stage. Juveniles in grow-out ponds were fed with a commercial pellet containing 37% crude protein, 6% crude fat, 2% crude fiber, 2% calcium, 1% total phosphorus, 1.8% lysine and 0.6% methionine. Feeding volume accounted for 2–3% of body weight, which were fed twice a day. After 95–109 days communally rearing the two generations (Table 4), a total of 25,212 individuals were harvested over the two generations (S1 Table).

All the surviving prawns were sampled, and for each of them the body weight, sex, family VIE code, pond, and harvest date were recorded. The phenotypes of the male prawns were classified. The claw of the male prawns contributes an important proportion of body weight. So, the male prawns with zero, one, or two claws were recorded as M0C, M1C, and M2C, respectively. Reproductive status is sometimes used as a common classification criterion for female prawns. While, the results of Hung et al. [26] suggested that body weights of female reproductive statuses were essentially the same traits and they could be analyzed together. This analytical model has also been used in many studies of *M*. *rosenbergii* [3, 7]. So the females were not classified in this study. Individuals which were above average body weight and healthy, were transferred to a cuboidal net cage with a side length of 0.25 m until they were transferred to the 3 m^2^ tanks for fertilization. A four-digit code label was placed above the cage to distinguish different individuals.

### Data analysis

The results of the significance test of fixed effects and covariate (age) are shown in Table 6. All the main effects, the two-way interactions and covariates among them were statistically significant for all individuals (*P* < 0.001), except for interaction between generation and pond (*P* > 0.05), which was statistically significant for each sex (*P* < 0.001). Within each sex, different effects showed different degrees of influence, which was mostly because the classification criterions of the two sexes were different.

**Table 6:** Analysis of variance of harvest body weight (HBW) across generations: Test of fixed effects according to different sexes using ASReml 4.

Effect	All		Male		Female	
	*F* value	Prob. >*F*	*F* value	Prob. >*F*	*F* value	Prob. >*F*
Generation	1189.74	<0.001	1364.19	<0.001	380.05	<0.001
SexID	9615.77	<0.001	-	-	-	-
SexID.SexSpec	250.26	<0.001	91.23	<0.001	-	-
Generation.SexID.SexSpec	619.70	<0.001	1.69	0.166	-	-
Generation.PondID	2.06	0.128	43.12	<0.001	67.06	<0.001
Generation.PondID.SexID.SexSpe	21.84	<0.001	0.78	0.566	35.08	<0.001
Generation.PondID.SexID.SexSpe.Age	110.66	<0.001	19.85	<0.001	-	-

When estimating variance components and genetic correlations between sexes, the reconstructed and unreconstructed pedigrees were used at the same time for comparisons. When calculating selective responses and making the mating design, only reconstructed pedigree analysis results were used.

### Variance components and heritability estimation

Age at harvest was linearly related to HBW. Therefore, age was used as a covariate in the model. The variance components of HBW were evaluated with a restricted maximum likelihood (REML) approach in ASReml 4 [27]. The animal model was as follows:

$$\begin{array}{l} y_{ijklmn}=\mu +Gen_{i}+Sex_{j}+Sex_{j}*Spec_{k}+Gen_{i}*Sex_{j}*Spec_{k}+Gen_{i}*Pon_{l}+\\ Gen_{i}*Sex_{j}*Spec_{k}*Pon_{l}+Age_{m}(Gen_{i}*Sex_{j}*Spec_{k}*Pon_{l})+a_{m}+f_{n}+e_{ijklmn} \end{array}$$ (1)

where *y*_*ijklmn*_ is the observed HBW of the *m*th individual of the breeding population; *μ* is the overall mean; *Gen*_*i*_ (the *i*th generation), *Sex*_*j*_ (the *j*th gender), their interactions with *Spec*_*k*_ (the *k*th sex specific phenotype, including M0C, M1C, M2C in males), and *Pon*_*l*_ (the *l*th pond) (*Sex*_*j*_ * *Spec*_*k*_, *Gen*_*i*_ * *Sex*_*j*_ * *Spec*_*k*_, *Gen*_*i*_ * *Pon*_*l*_, *Gen*_*i*_ *Sex_*j*_ * *Spec*_*k*_ * *Pon*_*l*_) are fitted as fixed effects among generations; *Age*_*m*_(*Gen*_*i*_ * *Sex*_*j*_ * *Spec*_*k*_ * *Pon*_*l*_) is a linear covariate nested within the interaction among generation, sex and pond; *a*_*m*_ is the additive genetic effect of the *m*th prawn, *a*~(0,$\text{A}\sigma_{\text{a}}^{2}$), where A is the additive genetic relationship matrix among all prawns, including the parent prawns of the G_0_ generation; *f*_*n*_ is the random effect of the *n*th full-sib family, which is caused by the separate rearing of the full-sib families before communal rearing, *f*~(0,$\text{I}\sigma_{\text{c}}^{2}$), where I is an identity matrix; *e*_*ijklmn*_ is the random residual error of the *m*th individual, *e*~(0, $\text{I}\sigma_{\text{e}}^{2}$). For within-generation analysis, the generation effect was excluded from the model. In the G_0_ generation, the common environmental effect was removed from the model because of the convergence problem.

The genetic parameters before and after the pedigree reconstruction were estimated. The accuracy (*r*) of estimated breeding values before and after pedigree reconstruction was calculated using the following formula [28]:

$$r=\sqrt{1-\frac{s_{m}^{2}}{(1+f_{m})\sigma_{A}^{2}}}$$

Where *s*_*m*_ is the standard error of the *m*th individual, *f*_*m*_ is the inbreeding coefficient of the *m*th individual, and $\sigma_{A}^{2}$ is the additive genetic variance obtained from the mixed model.

### Genetic correlations between sexes

SPSS version 19.0 software was used to analyze the significant difference in HBW between males and females. Means of HBW between males and females within each generation were examined by an independent sample *t*-test.

A bivariate analysis model was used to evaluate the genetic correlations between males and females within each generation, where the male and female body weights were regarded as two different traits. The bivariate analysis model was the same as the univariate model, the year and sex effects were excluded:

$$y_{klmn}=\mu +Spec_{k}+Spec_{k}*Pon_{l}+Age_{m}(Spec_{k}*Pon_{l})+a_{m}+f_{n}+e_{klmn}$$ (2)

where *y*_*klmn*_ is the observed HBW of the *m*th individual with male or female gender; other fixed and random effects were the same as those in formula 1.

The phenotypic variance ($\sigma_{\text{p}}^{2}$) is obtained by adding additive variance ($\sigma_{\text{a}}^{2}$), common environmental variance ($\sigma_{\text{c}}^{2}$), and residual variance ($\sigma_{\text{e}}^{2}$) together. All variance components were estimated using within- and across- generation datasets. A complete pedigree from G_0_ is available and used in all variance estimation. Heritability (*h*^2^) was obtained from the $\sigma_{\text{a}}^{2}$ divided by $\sigma_{\text{p}}^{2}$. The common environmental coefficient (*c*^2^) was obtained from the $\sigma_{\text{c}}^{2}$ divided by $\sigma_{\text{p}}^{2}$.

Z-score was utilized to detect if there was significant difference in the heritability between males and females, and if the genetic correlations between different sexes were significantly deviated from one [29]:

$$z=\frac{xi-xj}{\sqrt{\left(\sigma_{i}^{2}+\sigma_{j}^{2}\right)}}$$

Where *x*_*i*_ and *x*_*j*_ are the heritabilities estimated for males and females; *σ*_*i*_ and *σ*_*j*_ are their standard errors, respectively. When detecting whether an estimate is significantly deviated from one, *x*_*j*_ and *σ*_*j*_ are set to one and zero, respectively.

### Realized genetic gain estimation

Two methods were used to estimate the realized response. One method was calculated from the differences between the least squares means of the selected population HBW and that of control population HBW. The other method was calculated from the differences between the EBVs of the selected population HBW and that of control population HBW (Formula 1). The least squares means of the selected and control populations were evaluated by the following linear model:

$$\begin{array}{l} y_{ijklmn}=\mu +Pop_{i}+Sex_{j}+Pon_{l}+Sex_{j}*Spec_{k}+Sex_{j}*Spec_{k}*Pon_{l}+\\ Age_{m}(Sex_{j}*Spec_{k}*Pon_{l})+Pop_{i}*Fam_{n}+e_{ijklmn} \end{array}$$ (3)

Where *y*_*ijklmn*_ is the *m*th individual HBW in G_1_; *μ* is the overall mean; *Pop*_*i*_ (selected or control population), *Sex*_*j*_(the *j*th gender), *Pon*_*l*_ (the *l*th pond) and their interactions with *Spec*_*k*_ (the *k*th sex specific phenotype) (*Sex*_*j*_ * *Spec*_*k*_, *Sex*_*j*_ * *Spec*_*k*_ * *Pon*_*l*_) are fitted as fixed effects in G_1_, *Age*_*m*_(*Sex*_*j*_ * *Spec*_*k*_ * *Pon*_*l*_) is a linear covariate, which is nested in the interaction among sex, sex specific phenotype and pond; *Pop*_j_*Fam_*n*_ is the random effect of the *n*th family which is nested in the *j*th population; *e*_*ijklmn*_ is the random residual error of the *m*th individual.

### Predicted genetic gain estimation

Formula 1 was used to estimate the EBVs (Estimated Breeding Value) of all animals in G_0_ and G_1_ based on the best linear unbiased prediction (BLUP) by ASReml 4 software. The predicted genetic gain of HBW after one generation selection was achieved by calculating the difference of the average EBVs between G_1_ and G_0_ (including the control families in G_1_). The selection response was achieved by calculating the difference between the least squares means of the selected population HBW and that of the control population HBW in G_1_.

## Results

### Descriptive statistics

The observation numbers, simple means, minimum and maximum, standard deviation, and coefficients of variation (CVs) values for HBW in each generation are shown in Table 7. The mean body weight of the selected population of G_1_ was smaller than that of the base population, G_0_. The relatively lower stocking and harvest density and longer days for grow-out test in G_0_ may be responsible for this trend (Table 4). In G_1_, the mean and standard deviation of the selected population HBW were higher than that of the control population. The mean HBW in males was higher than that in females in each generation, ranging from 22.03% to 53.47%. The HBW CVs in the two generations were 29.54% to 33.07%, where males were also higher than females. The differences of HBW between males and females were significant in each generation (*P* < 0.01).

**Table 7:** The number, mean and standard deviation for HBW of individuals with different phenotypic characteristics, by sex, population, and generation.

Generation	Population	Sex	Category	Number	Percentage/%^a^	Mean (g)	Minimum (g)	Maximum(g)	Standard deviation	Coefficient variation (%)
G_0_	Base	All	-	8361	-	45.87	3.8	98.98	15.17	33.07
		Male	All	4112	-	55.74**	5.3	98.98	15.35	27.54
			M0C	108	2.62	50.72	6.9	89.1	15.94	31.43
			M1C	421	10.24	53.20	6.8	80.9	15.08	28.35
			M2C	3582	87.11	56.19	5.3	98.98	15.31	27.25
		Female	All	4249	-	36.32**	3.8	64.1	6.29	17.32
G_1_	Selection	All	-	14728	-	33.71	3.6	85	9.96	29.54
		Male	All	6903	-	37.28**	3.6	85	11.4	30.58
			M0C	462	6.69	32.09	3.6	59	11.18	34.84
			M1C	1518	21.99	35.28	4	70	11.23	31.83
			M2C	4907	71.09	38.39	3.9	85	11.27	29.36
		Female	All	7825	-	30.55**	3.9	68.9	7.15	23.40
	Control	All	-	2123	-	29.11	3.8	61.00	9.26	31.81
		Male	All	1051	-	31.38**	3.8	61	11.11	35.40
			M0C	82	7.80	25.97	4.4	55	11.47	44.17
			M1C	249	23.69	29.57	3.8	60.9	10.84	36.66
			M2C	715	68.03	32.61	3.9	61	10.94	33.55
		Female	All	1072	-	26.88**	5.7	49.30	6.52	23.25

** Estimate is significantly different from males and females within generation (*P* < 0.01) in *t*-test.

^a^ Percentage refers to the ratio of the number of individuals in the specific category to the total number of individuals in the same generation with the same gender.

In G_0_, the percentages of male specific characteristics were 2.62% for M0C (males with no claw), 10.24% for M1C (males with one claw), and 87.11% for M2C (males with two claws). In G_1_, the percentages of male specific characteristics were 6.69–7.80% for M0C, 21.99–23.69% for M1C, and 68.03–71.09% for M2C. A total of 22 males and 6 females had no record. For males, M2C had the highest mean body weight, followed by M1C and M0C.

### Variance components, heritability, and common environmental coefficient

The results based on reconstructed pedigrees were very close to the results of the unconstructed pedigree, and there was no significant difference (Table 7). The most obvious difference is that the variance components of females in G_0_ could be estimated with reconstructed pedigree but not by unconstructed pedigree. This was mostly because the pedigree reconstruction increased genetic linkages among some founders. The accuracy of estimated breeding values after and before pedigree reconstruction was 0.633 and 0.631, respectively. After pedigree reconstruction, the accuracy improved by 0.38%.

Variance components, heritability, and the common environmental effect for HBW were estimated within- and across- generations. As shown in Table 8, the estimated heritabilities within each generation (0.338 ± 0.049, 0.198 ± 0.080, respectively) were moderate to high, and significantly deviated from zero (*P* < 0.05). The common environmental coefficient of G_1_ was 0.055 ± 0.030. The common environmental variance in G_0_ was negligible. The estimated heritability for the cross-generation dataset was moderate (0.212 ± 0.049). The common environmental coefficient was a little larger (0.063 ± 0.017) than within generations.

**Table 8:** Variance components, heritability, and the common environmental effect of HBW.

	Generation	Sex	Variance components				Heritability	Common environment	Genetic correlation
			$\sigma_{a}^{2}$	$\sigma_{c}^{2}$	$\sigma_{e}^{2}$	$\sigma_{p}^{2}$	*h*^*2*^±*se*	*c*^*2*^±*se*	*r*_*g*_
Pedigree reconstruction	G_0_	All	37.975	-	74.474	112.450	0.338±0.049	-	
		Male	84.067	-	103.636	187.70	0.448±0.062	-	0.529±0.093
		Female	19.920	1.079	19.554	40.553	0.491±0.170	0.027±0.069	
	G_1_	All	13.388	3.706	50.711	67.805	0.198±0.080	0.055±0.030	
		Male	19.960	5.323	75.122	100.410	0.199±0.089	0.053±0.035	0.763±0.142
		Female	10.438	2.671	24.918	38.027	0.275±0.100	0.070±0.038	
	Across	All	17.844	5.277	61.009	84.131	0.212±0.049	0.063±0.017	
		Male	41.612	7.426	85.771	134.840	0.309±0.067	0.055±0.021	
		Female	10.890	3.755	24.482	39.127	0.278±0.067	0.096±0.024	
Pedigree unconstruction	G_0_	All	36.536	-	75.191	111.730	0.327±0.047	-	
		Male	81.907	-	104.718	186.620	0.439±0.061	-	0.533±0.091
		Female	21.294	-	18.865	40.159	0.530±0.068	-	
	G_1_	All	13.614	3.526	50.599	67.738	0.201±0.079	0.052±0.030	
		Male	20.430	5.026	74.88	100.340	0.204±0.088	0.050±0.034	0.766±0.138
		Female	10.518	2.583	24.878	37.979	0.277±0.099	0.068±0.038	
	Across	All	17.592	5.311	61.135	84.038	0.209±0.049	0.063±0.017	
		Male	41.014	7.543	86.085	134.640	0.305±0.067	0.056±0.021	
		Female	10.845	3.720	24.505	39.069	0.278±0.066	0.095±0.024	

The HBW heritabilities of male and female prawns were moderate to high, ranging from 0.199 ± 0.089 to 0.491 ± 0.170. For within generation datasets, the estimated heritabilities for female HBWs were higher than that for males. For across generation dataset, the estimated heritability for female HBWs were lower than that for males. No significant differences were obtained in the heritability between male and female prawns within- and across- generations (*P* > 0.05). The common environmental coefficients were larger in females than in males, from both within- and across- generations.

The genetic correlations between sexes were moderate to high within each generation (0.529 to 0.763). In G_0_, it was significantly different from 1 (z = -5.17, *P* < 0.01). In G_1_, it was not significantly different from 1 (z = -1.68, *P* > 0.05).

### Selection response

The least squares means and EBVs of HBW in selected and control populations were shown in Table 9. The realized genetic gains after carrying out the selection for one generation were 14.01% and 11.52% by the above two methods, respectively. The predicted genetic gains of HBW per generation were calculated from two sets of genetic parameters. Set 1 was acquired from the across-generation dataset, while Set 2 was acquired from the mean value of two within-generation datasets (Table 10).

**Table 9:** Estimates of realized genetic gain for HBW in each generation.

Generation	Population	Selection response 1			Selection response 2		
		Least squares mean (g)	Genetic gain (g)	Percentage^a^ (%)	EBV	Genetic gain (g)	Percentage^a^ (%)
G_0_	Base	46.08	-	-	-0.0021	-	-
G_1_	Selection	33.86	4.16	14.01	3.57	3.42	11.52
	Control	29.70	-	-	0.15	-	-

^a^ Percentage refers to the least squares means of HBW for the control population per generation.

**Table 10:** Estimates of predicted genetic gain for HBW using two sets of genetic parameters in each generation.

Generation	Breeding value	Selection response	
		Genetic gain (g)	Percentage^a^ (%)
Set 1: *h*^*2*^ = 0.212, *c*^*2*^ = 0.063			
G_0_	-0.0021		
G_1_	3.17	3.17	10.67
Set 2: *h*^*2*^ = 0.268, *c*^*2*^ = 0.028			
G_0_	-0.017		
G_1_	3.45	3.47	11.68

^a^ Percentage refers to actual units in relation to the least squares means of HBW for the control population in G_1_ (Table 9).

The mean breeding values in G_1_ were significantly increased compared to that in G_0_. After performing the selection for one generation, the genetic gains of HBW were 3.17 g and 3.47 g with parameters from Set 1 and Set 2, respectively. The percentage increases were 10.67% and 11.68%, respectively. The predicted genetic gains using Set 1 were a little lower than that predicted using Set 2.

## Discussion

### Heritability and common environmental coefficient

After pedigree reconstruction, the estimated variance components were very close to the results of the unconstructed pedigree and the accuracy of estimating breeding value has been improved 3.8%, probably because there was not enough genetic relationship among founders themselves. The meaning for pedigree reconstruction is it would play an important role in avoiding mating among full-sib individuals.

The heritability for HBW of *M*. *rosenbergii* across generations was 0.212 ± 0.049, which was similar to the estimate of 0.22 ± 0.056 in India [4]. However, it was higher than the estimates of 0.14–0.15 in Vietnam [6, 7] and 0.11 ± 0.08 in Thailand [29]. A previous selection of *M*. *rosenbergii* in China only achieved an HBW heritability estimation of 0.056, which was considered to be caused by low genetic variations of the foundations [3]. In this study, the base population was composed of four different strains from four different countries (China selected strain, Thailand, Burma, and Bengal populations), which could assemble greater genetic variations. The present data supported the opinion that low genetic variations of the foundations led to a low HBW heritability estimation of *M*. *rosenbergii* [3]. Besides, many other factors, such as different geographical populations, environmental conditions, and statistical models, would also affect the heritability estimation of the same species [4, 30].

In G_0_, the common environmental variances could not be successfully estimated. It was most probably because there were not enough genetic ties among families. Lack of the common environmental coefficient (*c*^*2*^) in the linear mixed model led to a higher heritability estimation in G_0_. *c*^*2*^ was small but significantly different from zero across generations, which showed that the period of family construction between generations had a certain effect on HBW (Table 8). Notably, the standard error of heritability and the common environmental coefficient were small, which was possibly because of the large number of testing individuals in each family. Another issue that needed to be addressed was that aquaculture conditions should be fixed annually to obtain more accurate testing and evaluation results. The density in G_0_ was very low (3.19–3.29 ind/m^2^), which was far below the production density. So we increased the density in G_1_ (9.75–14 ind/m^2^). This would affect the social structure to some extent which could also be seen from the proportion of different male morphology in the two generations, but the impact on the analysis results was very limited. We analyzed the correlation among the initial stocking body weight, harvested body weight and survival rate, and found the results were very close in the two generations. It showed that the differences of density had little effect on growth, survival and other traits (S1 Fig, S2 Table).

### Sexual differences of HBW

The mean HBWs of male and female *M*. *rosenbergii* were significantly different at each generation (Table 7). Males were 53.46% heavier than the females in G_0_ and 19.39% heavier in G_1_ (an average result from both selected and control populations). Previous studies also reported that male *M*. *rosenbergii* were much heavier, that is 62% [4] or 93% [6] higher than female prawns. Sexual size dimorphism is a common phenomenon in crustaceans, like *Penaeus monodon*, *Fenneropenaeus chinensis*, and *Litopenaeus vannamei* [31–33]. In *M*. *rosenbergii*, the average HBW of males is higher than that of females, which might be because females would allocate lots of energy to ovarian development and incubation [34, 35]. The HBW CVs were 27.54–35.40% in males and 17.32–23.40% in females, which were similar to values reported by Luan, et al. (20.58–50.13% in males and 6.57–34.54% in females) [3], though lower than those reported by Pillai, et al. (51% in males and 71% in females) [4]. The relatively lower HBW CVs is due to a relatively longer grow out period of *M*. *rosenbergii* used in the present study.

The HBW heritability of female *M*. *rosenbergii* (0.278 ± 0.067) was lower than that of males (0.309 ± 0.067) although there were no significant difference. This is different from previous studies in *M*. *rosenbergii* [3, 4, 6, 7, 29], which was most probably because the male prawns were classified in the genetic analysis, while female prawns did not. When the male and female HBWs were treated as separate traits, the genetic correlation was positive (0.529 ± 0.093 to 0.763 ± 0.142) and significantly different from one in the additional bivariate model, indicating that gender-related genes may have an effect on HBW. In addition, other factors such as behavioral factors, social interactions, and food deprivation might also affect the sexual dimorphism of HBW heritability [36].

The classification of sex related characteristics in *M*. *rosenbergii* has been reported in many studies. For males, the most common classification is divided into five distinct morphotypes: small claw, orange claw, blue claw, old blue claw, and no claw males, according to the claw color and the body size [37–41]. Genetic parameter evaluations of *M*. *rosenbergii* breeding populations were often based on the above classification [4, 6, 7, 41]. Notably, according to our preliminary statistics, the claw weight of male *M*. *rosenbergii* was very high, and one claw accounted for about 10% of HBW. Males often face intensive social interactions in the process of communally rearing, which would lead to the loss of one or two claws. This will greatly affect the final harvest weights. In this study, one claw and no claw males accounted for 23.53% of the total number (Table 7). Therefore, the claw number was used as a classification method for males in this study.

### Selection response

Although Gjedrem [42] summarized that the genetic gain for one generation was about 10% to 20% for aquatic animal species, the reported selection response of HBW of *M*. *rosenbergii* was not that high. In Vietnam, the selection response of HBW of *M*. *rosenbergii* reached an average rate of about 7% per generation [6, 7]. In China, it was 6.56% per generation [3]. In the present breeding program, the realized response reached 14.01% or 11.52% after performing one generation selection, almost two times that of previous breeding programs. The predicted response was also over 10% after performing one selection. The obvious genetic progress is likely to benefit from the great genetic variation, which is the most important factor to determine the genetic progress of breeding objectives. Moreover, the optimum contribution selection method might also play an important role. Selecting and mating parents is quite important for a breeding program. The optimum contribution selection method provides a powerful tool to establish equilibrium between the genetic gains of the next generation and limit the inbreeding rate by restricting the increase in average co-ancestry [43]. This is as optimal contribution selection puts more selection pressure on Mendelian sampling term over truncated selection [44–46]. Additionally, selection theory points out that sustained genetic progress was from the creation of a covariance between Mendelian sampling terms and the long-term genetic progress of candidate parents [47, 48]. The optimum contribution selection is more applied in terrestrial animals [49–52]. In aquatic animals, most studies have been carried out based on simulation and practical breeding projects [53–55].

In the present breeding program, increased density in G_1_ would result in more social interaction, and due to the suppression of growth via social dominance, estimates of realized genetic gain based on least squares mean was a little higher than that based on breeding value and predicted genetic gain. The good consistency between them indicated that the current genetic information could exactly partition the additive genetic variance. Our results show that the optimal genetic contribution selection can also be used as an effective breeding strategy in aquatic animals.

## Supporting information

S1 Fig Analysis of correlation between initial weight, harvested weight and survival rate of families. (DOCX)

S1 Table The individual data for the analysis in the present study. (XLSX)

S2 Table Information of stocking body weight, harvest body weight and survival rate of each family. (XLSX)
