# Supplementary material for: Genetic parameters and selection response for the harvest body weight of the giant freshwater prawn (Macrobrachium rosenbergii) in a breeding program in China
Source: PLoS One. 2019 Aug 12;14(8):e0218379. doi: 10.1371/journal.pone.0218379 (PMC6690521; doi:10.1371/journal.pone.0218379)
Supplement: S1 Fig — (DOCX) [file pone.0218379.s001.docx]

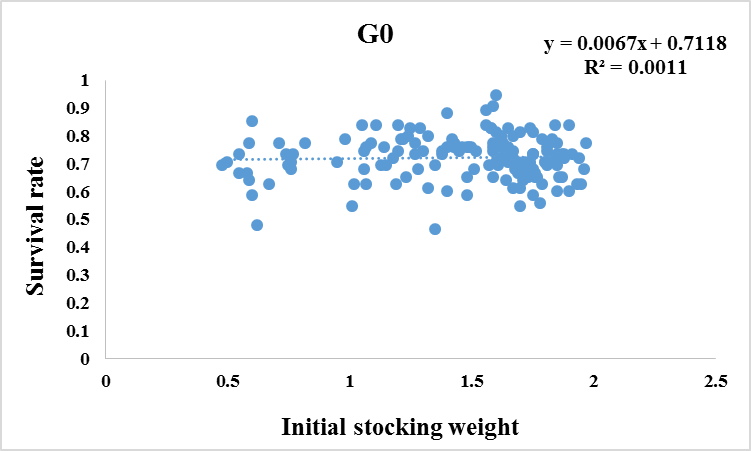

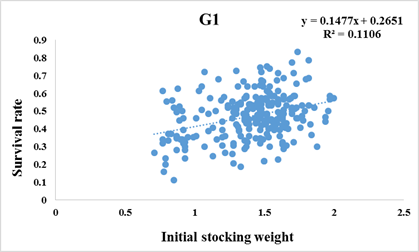


Fig 1. Analysis of correlation between initial stocking body weight and survival rate.


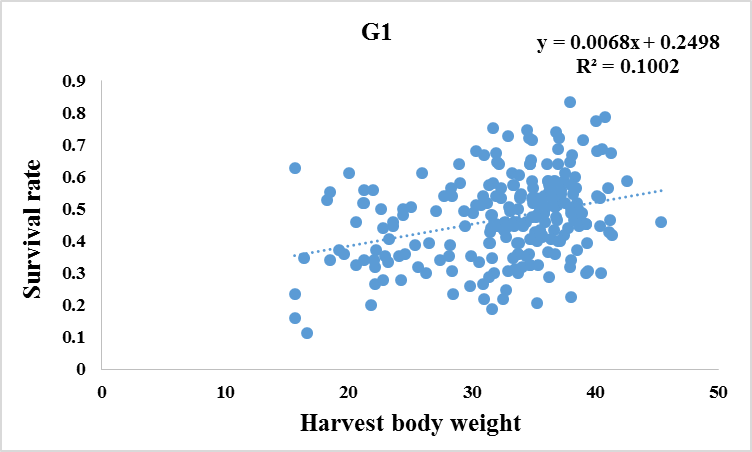

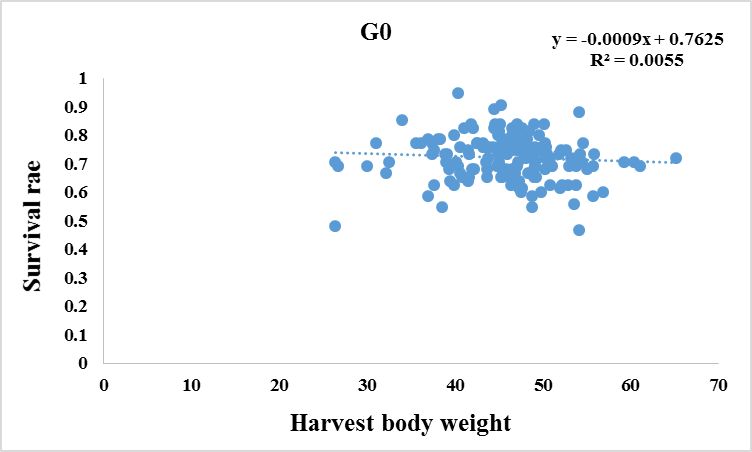


Fig 2. Analysis of correlation between harvest body weight and survival rate.
